# Supplementary figures and images for: Polystyrene microplastic particles induce endothelial activation
Source: PLoS One. 2021 Nov 17;16(11):e0260181. doi: 10.1371/journal.pone.0260181 (PMC8598073; doi:10.1371/journal.pone.0260181)

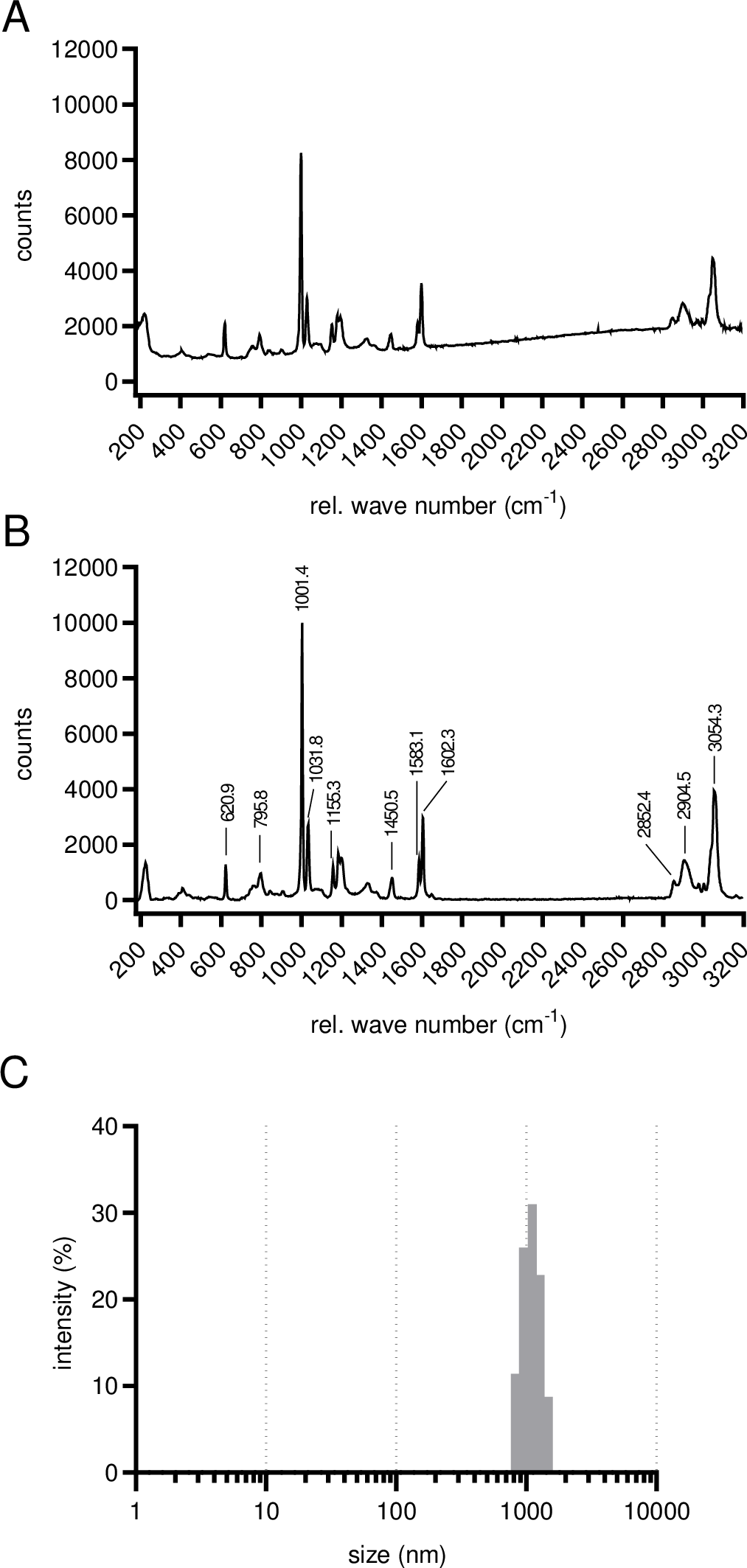

Supplement: S1 Fig — (A) Raman spectrum of PS particles used in this study (B) Raman spectrum of PS particles as a reference, obtained at ramanlife.com. (C) Size distribution of PS particles were assessed by dynamic light scattering and presented as percent intensity. (TIF) [file pone.0260181.s001.tif]

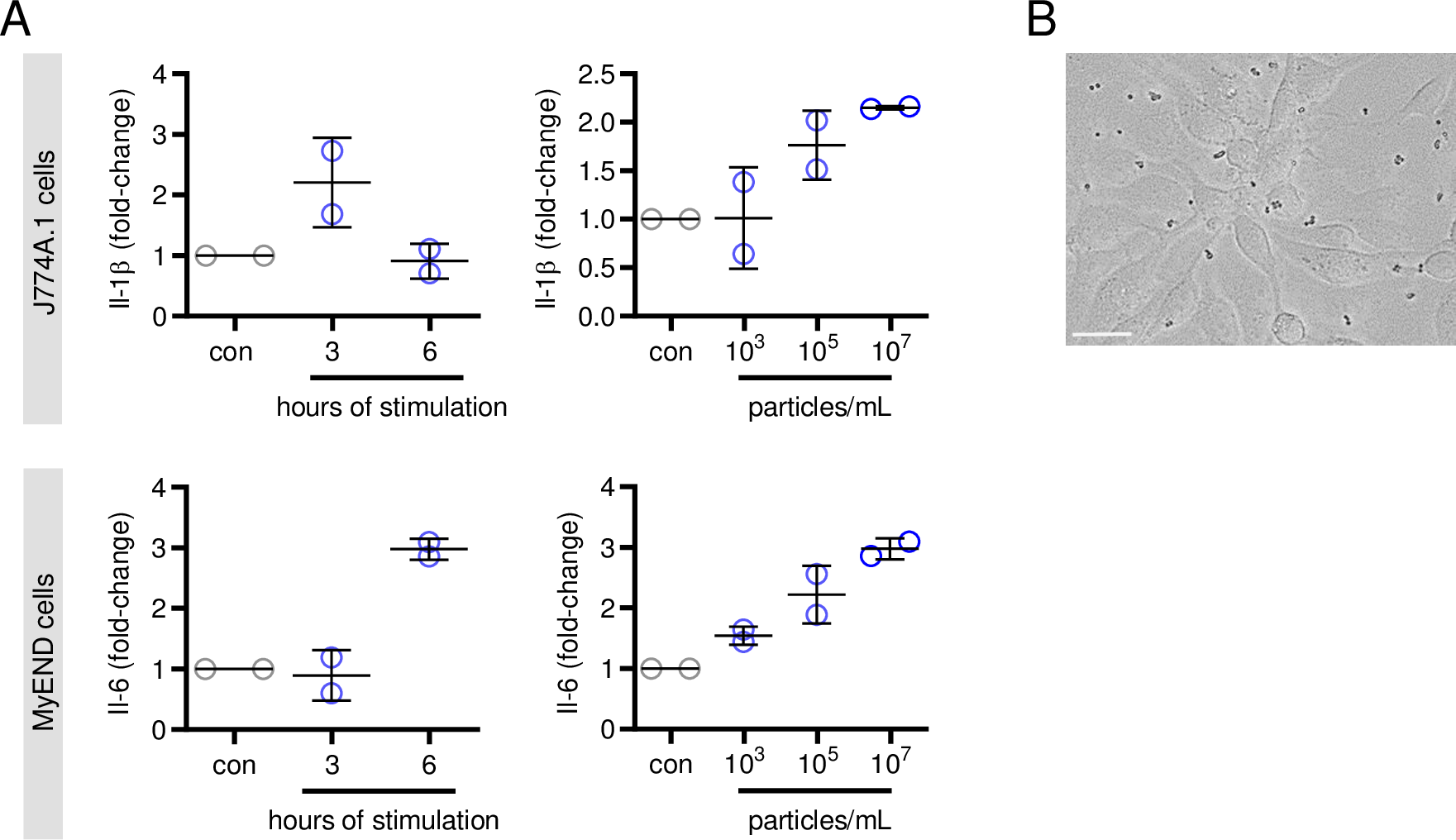

Supplement: S2 Fig — (A) Pilot study experiments (n = 2) in J774A.1 (top) and MyEND (bottom) cells using different time points (3 and 6 hours with a concentration of 107 particles/mL) and concentrations of PS particles (103, 105 and 107 particles/mL for 3 hours in J774.A1 cells and 6 hours in MyEND cells) to investigate kinetic and dosage effects. (B) Representative brightfield microscopy image showing a MyEND cell monolayer stimulated with PS particles at a concentration of 107 particles/mL. Scale bar = 20 μm. (TIF) [file pone.0260181.s002.tif]
