# Supplementary material for: Polystyrene microplastic particles induce endothelial activation
Source: PLoS One. 2021 Nov 17;16(11):e0260181. doi: 10.1371/journal.pone.0260181 (PMC8598073; doi:10.1371/journal.pone.0260181)
Supplement: Attachment — Submitted filename: Response to Reviewers.docx [file pone.0260181.s005.docx]

**Reviewer #1**

We thank Reviewer#1 to further improve the quality of our manuscript. All corrections are highlighted in red color in the revised version of our manuscript.

*Reviewer:* First of all, in my opinion, even define plastic as a "resource" is somehow incorrect. Therefore, the authors have to review the first sentence of the abstract. Plastic is a material/a product....
*Authors:* That is true, mineral oil would be the raw material in this context. We have decided to use the term material (line 30).

*Reviewer:* "In vivo" and "in vitro" must be written to Italic character.

*Authors:* We have now italicized both terms throughout the manuscript.

*Reviewer:* Check References.

*Authors:* We have checked again the correct use of all references.
